# OSTEOPAThic Health outcomes In Chronic low back pain: The OSTEOPATHIC Trial

**DOI:** 10.1186/1750-4732-2-5

**Published:** 2008-04-25

**Authors:** John C Licciardone, Hollis H King, Kendi L Hensel, Daniel G Williams

**Affiliations:** 1 The Osteopathic Research Center, University of North Texas Health Science Center-Texas College of Osteopathic Medicine, Fort Worth, TX 76107, USA

## Abstract

**Background:**

Osteopathic manipulative treatment (OMT) and ultrasound physical therapy (UPT) are commonly used for chronic low back pain. Although there is evidence from a systematic review and meta-analysis that OMT generally reduces low back pain, there are no large clinical trials that specifically assess OMT efficacy in chronic low back pain. Similarly, there is a lack of evidence involving UPT for chronic low back pain.

**Methods:**

The OSTEOPAThic Health outcomes In Chronic low back pain (OSTEOPATHIC) Trial is a Phase III randomized controlled trial that seeks to study 488 subjects between August 2006 and June 2010. It uses a 2 × 2 factorial design to independently assess the efficacy of OMT and UPT for chronic low back pain. The primary outcome is a visual analogue scale score for pain. Secondary outcomes include back-specific functioning, generic health, work disability, and satisfaction with back care.

**Conclusion:**

This randomized controlled trial will potentially be the largest involving OMT. It will provide long awaited data on the efficacy of OMT and UPT for chronic low back pain.

**Trial registration:**

, NCT00315120

## Background

Low back pain was the most common reason for office visits to osteopathic physicians in the 1977–1978 National Ambulatory Medical Care Surveys [1]. More contemporary national surveys have shown that a majority of patients who visit osteopathic physicians continue to report receiving treatment for musculoskeletal disorders [2], including osteopathic manipulative treatment (OMT) [3]. Osteopathic physicians play a unique role in treating patients with low back pain in the United States because they may provide OMT in addition to or instead of conventional medical treatment [4].

Osteopathic treatment of low back pain is based on four key principles [5]: (1) the body is a unit; (2) the body possesses self-regulatory mechanisms; (3) structure and function are reciprocally interrelated; and (4) rational therapy is based on an understanding of body unity, self-regulatory mechanisms, and the interrelationship of structure and function. Several randomized clinical trials of OMT for low back pain have been conducted [6-13]. These all involved subjects in ambulatory settings; however, they included relatively small numbers of subjects and were characterized by variations in methodology and outcomes among the trials. A meta-analysis of relevant data from these trials found that subjects who received OMT experienced significantly greater pain reduction than subjects who received control treatments [14]. Nevertheless, commentators continue to call for sufficiently powered trials to assess the efficacy of OMT for low back pain [15].

Ultrasound physical therapy (UPT), often termed "therapeutic ultrasound," is a commonly used modality for treatment of low back pain, with physical therapists reporting use in 60% [16] to 80% [17] of cases. Ultrasound physical therapy consists of inaudible acoustic vibrations delivered at a frequency between 0.75 and 3.0 MHz and intensity between 0.5 and 3 W/cm^2 ^[18,19]. The lower-frequency sound waves penetrate into deeper tissues such as joints, muscles, and bones, and produce thermal effects that are not normally perceived by patients. There is evidence that ultrasound accelerates tissue regeneration, increases pain thresholds, stimulates bone growth, and increases tendon extensibility [20]. As a deep-heating modality, UPT may ameliorate subacute or chronic soft-tissue inflammation via increased tissue temperature or blood flow [21].

The Philadelphia Panel Evidence-Based Clinical Practice Guidelines on Selected Rehabilitation Interventions for Low Back Pain found only one small study that addressed the efficacy of UPT for low back pain over 40 years ago [22]. This randomized controlled trial involving 36 subjects found no evidence of efficacy (i.e., pain reduction) after one month of treatment when comparing continuous ultrasound vs. placebo [23]. Consequently, the Philadelphia Panel concluded that there was poor evidence to include or exclude UPT alone as an intervention for chronic low back pain. More recently, the Working Group on Guidelines for Chronic Low Back Pain concluded that it could not recommend UPT as a treatment for chronic low back pain [24].

## Methods

### Subject recruitment

**Figure 1:**
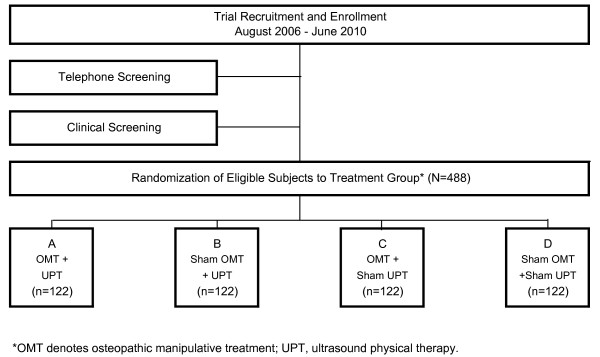
Flow of subjects from recruitment through randomization.

Subjects will be recruited by advertising in local newspapers and by seeking referrals from local physicians and from clinics affiliated with the University of North Texas Health Science Center. Subjects with constant or intermittent low back pain for at least three months will be sought. Terms other than "chronic low back pain" to be used in recruiting subjects will include "backache," "sciatica," and "lumbago." All subjects will be volunteers who are compensated for their time and travel at each study visit. Consent will be obtained from all subjects, and participants may withdraw at any time without penalty. The flow of subjects from recruitment through randomization is presented schematically in Figure 1.

### Sample size and statistical power

The primary outcome will be change in low back pain with OMT during the trial as measured by repeated visual analogue scale (VAS) scores for pain over 12 weeks. Based on recruitment and enrollment in the North Texas Chronic Low Back Pain Trial [12], 488 subjects was a feasible sample size target for the period August 2006 through June 2010. In a meta-analysis of the efficacy of OMT for low back pain, an effect size of 0.26 was observed for OMT vs. active treatment or placebo control [14]. Thus, using this assumed effect size, type I and type II error rates of 0.05 and 0.20, respectively, 244 subjects assigned to receive OMT (in either Group A or Group C) and 244 subjects assigned to receive sham OMT (in either Group B or Group D), the estimated statistical power of the trial for this pain outcome is 82%. For the secondary outcomes based on widely used research instruments (i.e., the Roland-Morris Disability Questionnaire [RMDQ] and the Medical Outcomes Study Short Form – 36 [SF-36] Health Survey), a total sample size of 488 will provide statistical power greater than 95% in detecting clinically relevant outcomes (a 2-point difference between groups on the RMDQ [25] and a 10-point difference between groups on the SF-36 general health scale [26]) with OMT.

### Inclusion criteria

To be eligible for consideration as a trial subject, a participant must meet all of the following telephone screening criteria: (1) be between 21 and 69 years of age; (2) give a positive response to the screening item, "Have you had low back pain constantly or on most days for the last three months?"; (3) identify low back as the primary site of pain; (4) agree to forego any type of extra-trial manipulation (either chiropractic or osteopathic) or physical therapy; (5) understand and complete trial questionnaires in English, or, if available, with appropriate translation services for other languages; (6) give written informed consent for clinical screening and, if selected, for trial participation; and (7) not be pregnant or plan to become pregnant during the course of the trial. Participants of childbearing potential will be required to have a negative urine pregnancy test and to be willing to maintain an acceptable method of contraception throughout the trial to be eligible for consideration as a subject. Pregnant women will be excluded from the study because back pain may resolve spontaneously after pregnancy and because the effects of UPT on the developing fetus are unknown.

### Exclusion criteria

The following criteria will be used during telephone screening to exclude potential subjects: (1) history of any of the following conditions which may be underlying causes of low back symptoms: (a) cancer; (b) spinal osteomyelitis; (c) spinal fracture; (d) herniated disc; (e) ankylosing spondylitis; (f) cauda equina syndrome; (2) history of surgery involving the low back within the past year or planned low back surgery in the future; (3) history of receiving Worker's Compensation benefits within the past three months; (4) involvement in current litigation relating to back problems; (5) current pregnancy or plan to become pregnant during the course of the trial; (6) any of the following that may limit a treatment provider's choice of OMT techniques or hamper a subject's compliance with the trial protocol: (a) angina or congestive heart failure symptoms that occur at rest or with minimal activity; or (b) history of a stroke or transient ischemic attack within the past year; (7) any of the following that may represent potential contraindications to receiving UPT: (a) implantation of a cardiac pacemaker; (b) implantation of artificial joints or other biomedical devices; (c) active bleeding or infection in the low back; or (d) pregnancy (8) use of intravenous, intramuscular, or oral corticosteroids within the past month; (9) history of chiropractic manipulation, OMT, or UPT within the past three months or on more than three occasions during the past year; or (10) being an osteopathic physician, allopathic physician, chiropractor, or physical therapist (or student of any of these professions).

### Clinical screening

A written informed consent will be administered to those participants who pass the initial telephone screening, and they will then proceed to the clinical screening. The clinical screenings will be performed by the same study personnel who provide the treatments. This clinical screening serves to independently confirm trial eligibility based on the telephone screening criteria described above, and provides an opportunity to clarify or update medical history information, if needed, or to perform any other necessary clinical examinations or tests to confirm continued trial eligibility.

Because about 12% of ambulatory patients with back pain have symptoms of sciatica or leg pain without neurological compromise related to lumbar disc herniation [27], such subjects will be included in this trial. However, to minimize the likelihood of including participants with a lumbar disc herniation, those with sciatica or leg pain will not be allowed to continue in the trial if they test positive for any of the following: (1) ankle dorsiflexion weakness; (2) great toe extensor weakness; (3) impaired ankle reflexes; (4) loss of light touch sensation in the medial, dorsal, and lateral aspects of the foot; (5) ipsilateral straight leg raising test (positive result: leg pain at <60 degrees); or (6) crossed straight leg raising test (positive result: reproduction of contralateral pain). These six neurological tests will allow detection of most clinically significant nerve root compromise due to L4-L5 or L5-S1 disc herniations, which together make up over 90% of all clinically significant radiculopathy attributable to lumbar disc herniations [27].

### Clinical trial protocol and timetable

**Figure 2:**
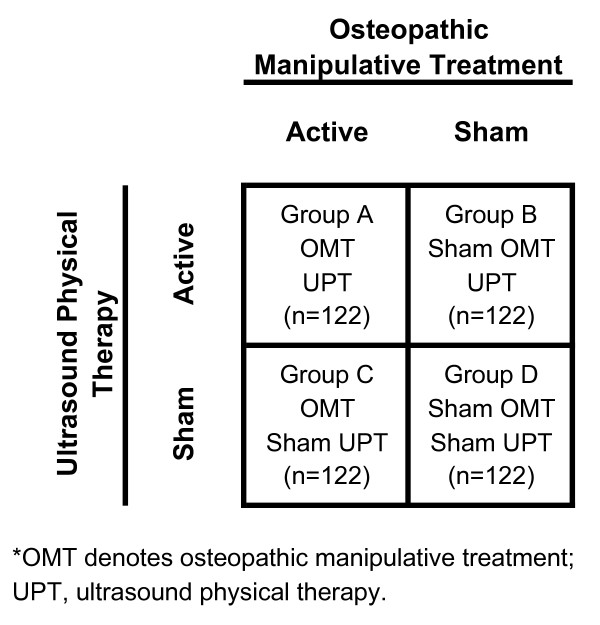
2 × 2 Factorial design.

Those subjects who pass the clinical screening process will return for a two-hour session for: (1) a baseline osteopathic history, examination, assessment, and treatment plan; (2) randomization to a treatment group; and (3) the initial (week 0) treatment session. Eligible subjects will be randomized to one of the four treatment groups, using a 2 × 2 factorial design as shown in Figure 2. Subjects in Group A will receive both active OMT and active UPT. Subjects in Group B will receive sham OMT and active UPT. Subjects in Group C will receive active OMT and sham UPT. Subjects in Group D will receive sham OMT and sham UPT. Blocked randomization will be performed throughout the trial to ensure that comparable numbers of subjects are assigned to each treatment group [28]. Subjects will be randomized in blocks of 24 (4 treatment groups × 6 subjects per treatment group) using computer generated assignments. The clinical trial coordinators who distribute and collect subject data forms at each study visit will be blinded to treatment allocation.

Additional follow-up treatments and data collection will occur during one-hour sessions, one, two, four, six, and eight weeks post-randomization. Exit data collection will be performed 12 weeks post-randomization. Treatment providers will be specialists (including affiliated practicing physicians), residents, or predoctoral fellows within the Department of Osteopathic Manipulative Medicine of the Texas College of Osteopathic Medicine at the University of North Texas Health Science Center. Subjects will be allowed to receive usual care from their personal health care providers. An overview of the clinical trial protocol and timetable is presented in Table 1.

**Table 1:** Clinical trial protocol and timetable

Task*	**Pre-Randomization**	**Weeks Post-Randomization**						
		
		**0**	**1**	**2**	**4**	**6**	**8**	**12**
Recruitment	●							
Telephone screening	●							
Clinical screening	●							
Data collection								
Sociodemographic data	●							
Visual analogue scale for low back pain	●	●	●	●	●	●	●	●
Roland-Morris Disability Questionnaire	●				●		●	●
Medical Outcomes Study Short Form – 36 Survey	●				●		●	●
Work disability measure	●				●		●	●
Patient satisfaction measure					●		●	●
OOSNF-History		●						
OOSNF-Examination		●						
OOSNF-Assessment and Plan		●	●	●	●	●	●	
Randomization		●						
Allocated treatment		●	●	●	●	●	●	

*OOSNF denotes Outpatient Osteopathic SOAP Note Form.

### Active treatments

#### Osteopathic manipulative treatment

Osteopathic practice entails a physician-patient interaction that is dynamic rather than static. Thus, OMT techniques should be individualized to the patient, and may need to be refined or changed over time based on the patient's response to OMT. This is entirely consistent with the view that different anatomical structures and physiological mechanisms may underlie pain in different patients with chronic low back pain. Ideally, practitioners should address how structure and function may affect low back pain and its progression, and then provide OMT by combining the most appropriate techniques from among the many available options [29].

Pragmatically, the trial protocol will be limited to the OMT techniques that are listed in the Glossary of Osteopathic Terminology [30]: articulatory treatment (ART), balanced ligamentous tension/ligamentous articular strain treatment (BLT), cranial treatment/osteopathy in the cranial field/cranial osteopathy (CR), counterstrain treatment (CS), direct treatment (DIR), facilitated positional release treatment (FPR), high velocity low amplitude (thrust) treatment (HVLA), indirect treatment (IND); integrated neuromusculoskeletal release (INR), ligamentous articular strain/balanced ligamentous tension treatment (LAS), muscle energy treatment (ME), myofascial release treatment (MFR), soft tissue treatment (ST), and visceral manipulative treatment (VIS). These 14 techniques include the vast majority of techniques used in patients with chronic low back pain.

In order to provide each subject with a comparable OMT intervention across providers, subjects will be evaluated at each encounter using the "dirty half dozen" framework [31]. This approach accepts that six entities are often encountered in patients with chronic low back pain: (1) non-neutral lumbar somatic dysfunction; (2) dysfunction of the symphysis pubis (pubic shear); (3) restriction of the anterior movement of the sacral base; (4) innominate shear dysfunction; (5) a short leg and pelvic tilt syndrome; and (6) muscular imbalance of the trunk and lower extremity (including psoas syndrome).

Subjects initially will be evaluated in the seated position for thoracic and lumbar somatic dysfunction. Screening in this position allows for not only tri-planar dysfunction diagnosis, but also for determination of areas of greater restriction, which may help the treatment provider determine where to initiate treatment within a given session. Key findings, including lumbar segmental dysfunction, non-neutral lumbar dysfunction, and lumbar muscle hypertonicity also will be graded as "0" for no dysfunction, "1" for mild to moderate dysfunction, and "2" for severe dysfunction. Subjects will be evaluated in the prone position for sacral landmarks and motion testing. This evaluation will assess sacral dysfunctions, including forward and backward sacral torsions, unilateral and bilateral flexion and extension lesions, and sacral shears. Sacral dysfunction will be graded on the same 0–2 scale described above for lumbar dysfunction. Subjects will be placed in left and right lateral recumbent positions for lumbar soft tissue, muscle energy, and high velocity low amplitude techniques. While in this position, sacral torsion muscle energy may be applied. Subjects will be placed in the supine position for pelvic landmark evaluation and motion testing. Particular attention will be paid to pubic dysfunction, iliac rotation, and iliac shears. Screening and treatment, if indicated, will be provided for psoas and anterior lumbar counterstrain points. Innominate dysfunction will be graded on the previously described 0–2 scale. Treatment providers will perform up to five additional OMT techniques based upon their evaluation of the subject and the subject's response to the standard protocol.

#### Ultrasound physical therapy

The UPT will include ultrasound as used in physical medicine and rehabilitation programs for a variety of musculoskeletal disorders [21,32]. The anatomical areas of the low back to be treated will be based on physical findings from the baseline osteopathic examination, assessment, and treatment plan. The UPT will be administered by the same providers who administer OMT. To achieve heating of deeper tissues, the UPT unit will be set to an intensity level of 1.2 W/cm^2^, using a frequency of 1 MHz. A 10 cm^2 ^applicator will be applied directly to the skin using a conductivity gel to enhance ultrasound absorption. The UPT intervention will last approximately 10 minutes, allowing the provider to treat about 150 to 200 cm^2 ^in the target treatment area and thereby produce deep muscle temperature elevations approaching 4°C. This heating effect may ameliorate symptoms of chronic pain and inflammation.

As there are no previous studies that have evaluated the combination of OMT and UPT, we will provide OMT prior to UPT to avoid potential difficulties in performing OMT after the administration of ultrasonic conductivity gel to the target treatment area. Subjects will be instructed to report any uncomfortable thermal (e.g., excessive heat or burning) or mechanical (e.g., excessive friction or trauma) effects at the treatment sites. Such reports will trigger treatment providers to reduce the intensity of the UPT to alleviate the reported discomfort or, if necessary, to discontinue the UPT for that session.

### Placebo control treatments

Sham OMT was adapted from the methodology established in the North Texas Chronic Low Back Pain Trial [12]. The treatment will include hand contact with minimal movement of body parts. The intent of the sham OMT is to apply manual forces of diminished magnitude purposely aimed to avoid treatable areas of somatic dysfunction relative to low back pain and, consequently, to minimize the likelihood of any therapeutic effect. Because sham OMT in the preliminary trial provided some therapeutic effect [12], the baseline osteopathic examination, assessment, and treatment plan will be used to guide the selection of anatomical regions for sham OMT, with the intent of avoiding those areas of somatic dysfunction that contribute most to the subject's low back pain.

Subjects assigned to receive sham OMT will be treated in positions similar to subjects receiving active OMT. Sham OMT subjects will receive several minutes of hands-on time in the seated position with attention paid to the thoracic and lumbar spine regions. Sham OMT subjects will also receive hands-on time in the prone and supine positions, including hands-on contact of the sacrum in the prone position and pelvic landmark examination in the supine position.

Sham UPT will be based on the methodology used in a previous trial of OMT in third-trimester pregnancy [33]. This treatment will provide tactile and manual stimulation of the low back and other anatomical areas that will  be treated with active or sham OMT. These anatomical areas will be based on findings from the baseline osteopathic examination, assessment, and treatment plan to maximize placebo credibility and expectations. Subtherapeutic ultrasound will be provided by setting the ultrasound unit to 0.1 W/cm^2 ^intensity at 1 MHz; however, the applicator head will be applied directly to the subject's skin in the low back using ultrasonic conductivity gel to simulate in all other respects the actual UPT maneuvers described above.

### Baseline data collection

Baseline data will be collected at the clinical screening visit prior to randomization. This includes sociodemographic data, insurance status, and a VAS for low back pain, which serves as the primary outcome measure. Other baseline measures include back-specific functioning (RMDQ), generic health status (SF-36), work disability, and back-specific satisfaction, as these are secondary outcome measures that address the four remaining recommended domains of patient-based outcomes in evaluating the treatment of spinal disorders [34]. Repeated measures of the primary and secondary outcome variables will be performed during the trial as indicated in Table 1. Additional data relevant to low back pain, medical comorbidities, and other co-treatments will also be collected.

#### Visual analogue scale

The VAS will consist of a 100-mm horizontal line labeled as "no pain" at its left end (measured as 0 mm) and as "worst possible pain" at its right end (measured as 100 mm). This measure is commonly used to assess changes in pain over time and has been recommended as an outcome measure in studies of spinal disorders such as low back pain [35]. It has been shown that data derived from such written scales among patients with chronic low back pain are normally distributed even when the scales are used without verbal instructions [35].

#### Roland-Morris Disability Questionnaire

Functional status and disability resulting from back pain will be measured with the RMDQ [36]. This questionnaire, which was derived from the Sickness Impact Profile, is short and simple to complete and appears to be well suited for studies involving patients with mild to moderate disability [37]. Empirical research suggests that the RMDQ poses less problems involving blank or multiple responses than either the Oswestry Disability Index or the Jan van Breemen Institute pain and functional capacity questionnaire and, therefore, may be the preferred instrument for assessing change over time in patients with low back pain [38]. The RMDQ consists of a series of 24 items. It is scored as the number of positive responses to these items, with higher scores reflecting greater functional disability.

#### Medical Outcomes Study Short Form – 36 Health Survey

The SF-36 will be used to measure generic health status. It is widely used for this purpose and has been used extensively in measuring clinical outcomes following medical interventions. The SF-36 provides data on health concepts using the following scales [26]: physical functioning, role limitations because of physical problems, bodily pain, general health, vitality, social functioning, role limitations because of emotional problems, and mental health. Construct validity of the SF-36 was established by factor analysis [39]. Studies including the internal consistency method, Cronbach's coefficient alpha, and test-retest coefficients support use of the SF-36 in detecting short-term changes in health, such as those being measured in this trial [26]. The reliability of the SF-36 has also been demonstrated by test-retest correlations based on intervals as long as six months between administrations [26]. Responses to each of the 36 survey items will be recorded and then standardized scores (ranging from 0 for worst possible health to 100 for best possible health) will be computed for each of the eight scales described above using the recommended algorithms. The SF-36 general health scale score will be the main focus of interest, as this represents a secondary outcome in the trial.

#### Work disability

Subjects who work will be asked to complete the following survey item: "During the past four weeks, how many days did back pain keep you from going to work?" A response section will allow for two digits and instructions will specify to record "00" if no work loss occurred.

#### Satisfaction with back care

Satisfaction with back care will be measured by responses to the following item: "Overall, how would you describe your satisfaction with the care for your back that you have received?" Likert-scale response options will include: "very satisfied," "satisfied," neither satisfied nor dissatisfied," "dissatisfied," or "very dissatisfied."

### Data management and analysis

The data will be entered into the SPSS for Windows software package (SPSS Inc, Chicago, IL) by independent research personnel using standardized formats and dual data entry to minimize keying errors. Statistical analyses will be performed using the intention-to-treat principle. Analysis of variance (ANOVA) will be the primary method of statistical analysis. This has the advantages of allowing for assessment of repeated outcome measures over time, interaction of the factors being studied, and control of important confounders if indicated. Missing values for outcome measures will be imputed using the last observation carried forward method.

## Ethical aspects

The study protocol was approved by the Institutional Review Board (IRB) of the University of North Texas Health Science Center prior to implementation. The IRB will continue to monitor the study regularly. Additionally, a Data Safety and Monitoring Board (DSMB) has been established, and will closely monitor the study for the occurrence of any serious adverse events. The study was registered as a Phase III trial with ClinicalTrials.gov in April 2006 (ClinicalTrials.gov identifier, NCT00315120).

## Conclusion

To the best of our knowledge, if successfully completed as described herein, the OSTEOPATHIC Trial will be the largest randomized controlled trial involving OMT. It will provide long awaited primary data on the efficacy of OMT and UPT for chronic low back pain.

## Competing interests

Dr. Licciardone is Editor-in-Chief of *Osteopathic Medicine and Primary Care*. He was not involved in the review of this manuscript or the editorial decision regarding its suitability for publication. The remaining authors declare that they have no competing interests.

## Authors' contributions

JCL conceived and designed the study, and acquired funding for it. He will be responsible for overall administration and direction of the project, analysis and interpretation of the data, and will give final approval of the manuscript to be published. HHK and KLH made substantial contributions to the development of the study protocol, particularly the active and sham treatments. They also provide subject treatments and conduct ongoing training for other treatment providers in the study protocol. DGW provides subject treatments. JCL was primarily involved in drafting the manuscript, with assistance from HHK, KLH, and DGW. All authors read and approved the final manuscript.
